# The effect of music therapy on delirium in patients receiving mechanical ventilatory support in the Intensive Care Unit: A protocol for systematic review and meta-analysis

**DOI:** 10.1097/MD.0000000000033956

**Published:** 2023-06-16

**Authors:** Changyan Zhou, Hong Ma, Xiaoxue Qi, Chunru Xu, Zina Liang

**Affiliations:** a Department of Critical Care Medicine, The Second Affiliated Hospital of Wenzhou Medical University, Wenzhou, China; b Child Intensive Care Unit, The Second Affiliated Hospital of Wenzhou Medical University, Wenzhou, China.

**Keywords:** delirium, intensive care unit, mechanical ventilatory support, meta-analysis, music therapy, protocol, systematic review

## Abstract

Delirium is a very common sick syndrome in patients receiving mechanical ventilatory support in the Intensive Care Unit (ICU). Music therapy is a promising non-pharmacological intervention. However, its effect on the duration, incidence and severity of delirium is unknown. So we will perform a systematic review and meta-analysis to evaluate the effect of music therapy on delirium in patients receiving mechanical ventilatory support in the ICU.

**Methods:**

This systematic review was registered in the PROSPERO. We will follow the Preferred Reporting Items for Systematic Reviews and Meta-analysis Protocol to accomplish the systematic review protocol. Searches of PubMed, EMbase, the Cochrane library, CBM, CNKI and Wanfang databases will be conducted through computer to collect randomized controlled trials (RCTs) on the effects of music therapy on delirium in patients receiving mechanical ventilatory support in the ICU. The search time will be all from database establishment to April 2023. Two evaluators will independently screen the literature, extract information and evaluate the risk of bias of included studies, then data analysis will be performed using Stata 14.0 software.

**Results:**

The results of this systematic review and meta-analysis will be publicly available and published in a peer-reviewed journal.

**Conclusion:**

This study will provide evidence-based medical evidence for music therapy to control delirium in patients receiving mechanical ventilatory support in the ICU.

## 1. Introduction

Delirium is an acute, reversible, widespread cognitive disorder psychotic syndrome characterized by fluctuating disorders of consciousness, inattention, disorganized thinking, or changes in level of consciousness.^[1,2]^ Patients receiving mechanical ventilatory support in the Intensive Care Unit (ICU) are at high risk of delirium.^[3]^ Delirium can be diagnosed when certain psychiatric symptoms are present without abnormal central nervous system function and organic comorbidities. And its main symptoms are relatively acute confusion, restlessness, excitement, hallucinations, and delusions.^[4]^ The literature reports that the incidence of delirium is generally 5% to 40%, up to 80%, and 50% to 60% in elderly patients.^[5]^ The risk of iatrogenic pneumonia increases tenfold after delirium, and the incidence of complications such as aspiration, pulmonary embolism, and pressure sores also increases greatly, which may cause accidental extubation, difficulty in weaning ventilators, or reintubation after extubation in mechanically ventilated patients.^[6]^ Active and effective nursing interventions by nursing staff for ICU patients are very important to avoid and reduce the occurrence of delirium in a timely manner, improve the prognosis of patients, and improve their quality of life.

The pathogenesis of delirium is still unclear, but nurses, as the staff with the longest contact time with ICU patients, have an important influence on the clinical care of delirious patients, and good nursing interventions can effectively improve the patient’s disease outcome and prognosis. Mechanically ventilated patients experience pain and anxiety, which are usually relieved by the tranquillizer independently associated with the onset of delirium.^[7–10]^ Despite the prevalence of delirium, there are no effective pharmacological interventions for delirium.^[11]^ Previous use of drugs such as haloperidol, ziprasidone, and acetylcholine-lowering drugs has not been superior to placebo.^[7]^ Therefore, non-pharmacological interventions may be potentially effective interventions for the treatment or prevention of delirium.^[12]^

Music therapy is a therapeutic modality in which medical psychology and music are interwoven. With the shift in medical model, music therapy is gradually being used in clinical practice.^[13]^ It has been reported in the literature that music therapy has a positive effect on preventing and reducing the occurrence of delirium.^[14]^ Music therapy affects the physiological functions of patients through music, promotes the release of beneficial chemicals such as acetylcholine, which can reduce the psychological reactions of anxiety, depression and fear, stabilize patients’ emotions, slow down their heart rate, dilate blood vessels, reduce cardiac load and enhance blood circulation, which play a role in regulating blood flow and nervous system functions.^[3,13,14]^

The ability of music therapy to improve delirium in patients receiving mechanical ventilatory support in the ICU is currently controversial.^[11,15–17]^ Therefore, we will perform a systematic review and meta-analysis to investigate the effect of music therapy on delirium in patients receiving mechanical ventilatory support in the ICU.

## 2. Methods

### 2.1. Protocol and registration

This systematic review was registered in the PROSPERO (registration number: CRD42023418303). We will follow the Preferred Reporting Items for Systematic Reviews and Meta-analysis Protocol to accomplish the systematic review protocol.^[18]^ This study is conducted for the secondary collection and analysis of original data; therefore, ethical approval is not required.

### 2.2. Type of study

A randomized controlled trial (RCT) incorporating the effects of music therapy on delirium in patients receiving mechanical ventilatory support in the ICU.

### 2.3. Types of participants

Age > 18 years, duration of mechanical ventilatory support in the ICU > 24h, consistent with the indications for the application of mechanical ventilatory support.

### 2.4. Interventions and comparators

The control group will be given routine ICU care, including basic care, intensive care, specialist care and health education; the experimental group will be given music therapy on the basis of routine ICU care.

### 2.5. Types of outcome measures

Incidence of delirium, duration of delirium state, severity of delirium, duration of mechanical ventilatory support, length of ICU stay, and in-hospital mortality.

### 2.6. Search strategy

Computer search of PubMed, EMbase, the Cochrane library, CBM, CNKI, and Wanfang databases will be conducted to collect RCTs on the effect of music therapy on delirium in patients receiving mechanical ventilatory support in the ICU. Only studies published in English and Chinese will be included. The searching strategy in PubMed is shown in Table 1.

**Table 1:** Search strategy for PubMed.

Number	Search terms
#1	Delirium[MeSH]
#2	Delirium of Mixed Origin[Title/Abstract]
#3	Subacute Delirium[Title/Abstract]
#4	Delirium, Subacute[Title/Abstract]
#5	Deliriums, Subacute[Title/Abstract]
#6	Mixed Origin Delirium[Title/Abstract]
#7	Mixed Origin Deliriums[Title/Abstract]
#8	Subacute Deliriums[Title/Abstract]
#9	OR/1-8
#10	Intensive Care Units[MeSH]
#11	Care Unit, Intensive[Title/Abstract]
#12	Care Units, Intensive[Title/Abstract]
#13	Intensive Care Unit[Title/Abstract]
#14	Unit, Intensive Care[Title/Abstract]
#15	Units, Intensive Care[Title/Abstract]
#16	OR/10-15
#17	Music Therapy[MeSH]
#18	Therapy, Music[Title/Abstract]
#19	Music[Title/Abstract]
#20	OR/17-19
#21	Randomized Controlled Trial [Publication Type]
#22	Randomized Controlled Trial[MeSH]
#23	Random*[Title/Abstract]
#24	OR/21-23
#25	#9 AND #16 AND #20 AND #24

### 2.7. Data collection and analysis

#### 2.7.1. Selection of studies.

**Figure 1.**
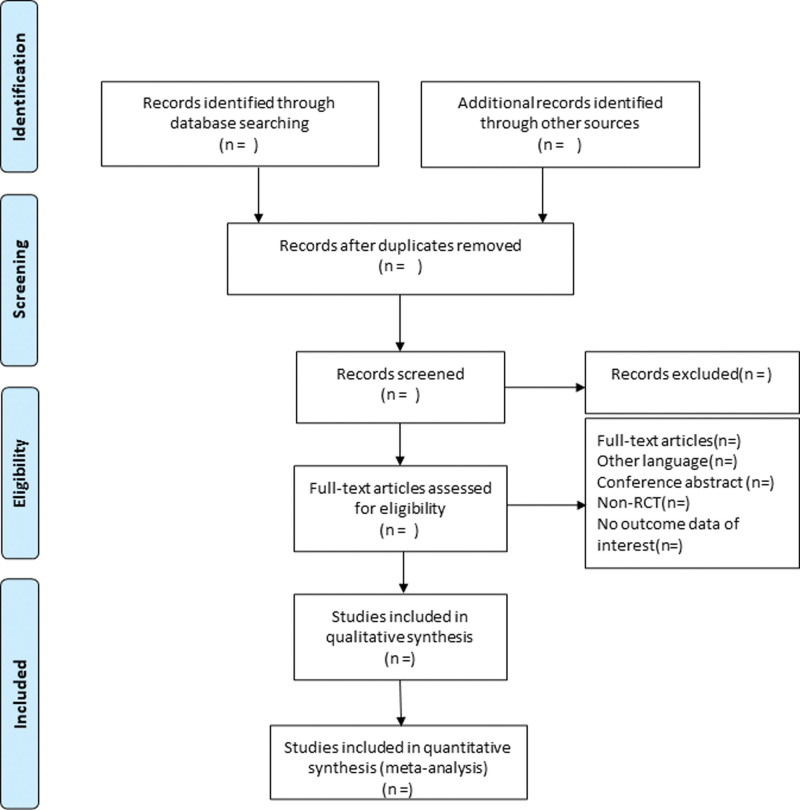
Flow diagram of study selection process. RCTs = randomized controlled trial.

Based on the inclusion and exclusion criteria, 2 investigators will independently read the titles and abstracts of the obtained literature, and after excluding trials not meeting the inclusion criteria, the remaining literature will be read in full, screened, and cross-checked. In case of disagreement, the discussion will help to resolve it. The procedures of study selection will be performed in accordance with the Preferred Reporting Items for Systematic reviews and Meta-Analysis flow chart (as shown in Fig. 1).

#### 2.7.2. Data extraction and management.

Two researchers will independently screen the literature, extract and cross-check the data. In case of disagreement, discussion will be held to help resolve it. Data extraction will be included: disease type, date of publication, age, sex, study site, sample size, intervention, follow-up time, and mean and standard deviation of continuous indicators for outcome indicators.

#### 2.7.3. Risk of bias assessment.

The quality of the included literature will be independently evaluated independently by 2 investigators, and the results will be cross-checked. The risk of bias will be assessed based on the risk of bias assessment tool for RCTs in the Cochrane Handbook,^[19]^ including the generation of random sequences, allocation concealment, implementation of blinding, completeness of outcome data, selective reporting of results, and other biases.

#### 2.7.4. Measures of treatment effect.

Stata 14.0 software will be used to calculate the outcome data. We will use 95% confidence intervals and risk ratios to estimate binary data. For continuous outcomes, comparisons will be made using weighted mean differences or standardized mean differences.

#### 2.7.5. Additions of missing data.

If an article has any missing or insufficient data, we will send basic information to the relevant authors via email. If no contact is established or the data is incomplete, the study will be excluded.

#### 2.7.6. Assessment of heterogeneity and data synthesis.

The heterogeneity of included RCTs will be assessed by the Chi-square test. If *I*^2^ ≤ 50% and *P* ≥ .10, a fixed-effects model will be adopted; Otherwise, a random-effects model will be used for analysis.

### 2.8. Subgroup analysis

If there is significant heterogeneity in the study data, subgroup analyses will be conducted according to different types of music therapy, different control groups, and duration of intervention.

### 2.9. Sensitivity analysis

After excluding the low-quality studies, the combined effect sizes will be re-estimated and compared with the results of the meta-analysis before exclusion, thus exploring the effect of the remaining RCTs on the combined effect size and the robustness of the results.

### 2.10. Reporting bias analysis

When more than 10 studies are included, funnel plots will be depicted to detect publication bias.^[20–22]^

### 2.11. Confidence in cumulative evidence

The Grading of Recommendations Assessment, Development, and Evaluation method was conducted to assess the quality of evidence for each outcome.^[23]^ The quality of evidence will be classified as high, medium, low, or very low.

### 2.12. Ethics and dissemination

Ethics approval is not required because this is conducted on published data and not on individual patient information. The results will be submitted to a peer-reviewed journal and presented at relevant conferences.

### 2.13. Amendments

The information will be described in the final report, and if the protocol is modified.

## 3. Discussion

Delirium is an acute brain failure syndrome, and patients receiving mechanical ventilatory support are at high risk of delirium.^[24]^ Intubated patients experiencing pain, anxiety, and physiological stress often require treatment with tranquillizer, which are also risk factors for the occurrence of delirium, and in turn, a vicious cycle of pain, anxiety, sedation, and delirium.^[25]^ Therefore, how to effectively prevent and reduce the incidence of delirium has become a hot topic of clinical research. Previously published RCTs on the effects of music therapy on delirium in patients receiving mechanical ventilatory support in the ICU have reached different conclusions^[11,15–17]^caused by that these studies were limited by small sample sizes. To overcome these limitations, we will perform a high-quality systematic review and meta-analysis to assess the effect of music therapy on delirium in patients receiving mechanical ventilatory support in the ICU.

## Author contributions

**Conceptualization:** Zina Liang, Changyan Zhou.

**Data curation:** Changyan Zhou.

**Formal analysis:** Changyan Zhou.

**Funding acquisition:** Zina Liang.

**Investigation:** Hong Ma.

**Methodology:** Hong Ma.

**Project administration:** Zina Liang.

**Resources:** Hong Ma.

**Software:** Hong Ma, Xiaoxue Qi, Chunru Xu.

**Supervision:** Zina Liang.

**Validation:** Xiaoxue Qi, Chunru Xu.

**Visualization:** Xiaoxue Qi, Chunru Xu.

**Writing – original draft:** Zina Liang, Changyan Zhou.

**Writing – review & editing:** Zina Liang, Changyan Zhou.
